# The *vicK* gene of *Streptococcus mutans* mediates its cariogenicity via exopolysaccharides metabolism

**DOI:** 10.1038/s41368-021-00149-x

**Published:** 2021-12-16

**Authors:** Yalan Deng, Yingming Yang, Bin Zhang, Hong Chen, Yangyu Lu, Shirui Ren, Lei Lei, Tao Hu

**Affiliations:** State Key Laboratory of Oral Diseases & National Clinical Research Center for Oral Diseases & Department of Preventive Dentistry, West China Hospital of Stomatology, Sichuan University, 610041 Chengdu, China

**Keywords:** Dental caries, Biofilms

## Abstract

*Streptococcus mutans* (*S. mutans*) is generally regarded as a major contributor to dental caries because of its ability to synthesize extracellular polysaccharides (EPS) that aid in the formation of plaque biofilm. The VicRKX system of *S. mutans* plays an important role in biofilm formation. The aim of this study was to investigate the effects of *vicK* gene on specific characteristics of EPS in *S. mutans* biofilm. We constructed single-species biofilms formed by different mutants of *vicK* gene. Production and distribution of EPS were detected through atomic force microscopy, scanning electron microscopy and confocal laser scanning microscopy. Microcosmic structures of EPS were analyzed by gel permeation chromatography and gas chromatography-mass spectrometry. Cariogenicity of the *vicK* mutant was assessed in a specific pathogen-free rat model. Transcriptional levels of cariogenicity-associated genes were confirmed by quantitative real-time polymerase chain reaction. The results showed that deletion of *vicK* gene suppressed biofilm formation as well as EPS production, and EPS were synthesized mostly around the cells. Molecular weight and monosaccharide components underwent evident alterations. Biofilms formed in vivo were sparse and contributed a decreased degree of caries. Moreover, expressional levels of genes related to EPS synthesis were down-regulated, except for *gtfB*. Our report demonstrates that *vicK* gene enhances biofilm formation and subsequent caries development. And this may due to its regulations on EPS metabolism, like synthesis or microcosmic features of EPS. This study suggests that *vicK* gene and EPS can be considered as promising targets to modulate dental caries.

## Introduction

Dental caries is a chronic infectious disease occurring on mineralized tooth tissue, which is considered one of the most common health problems globally in 2015 posing considerable economic, social, and health-related burden.^1–3^ *Streptococcus mutans* (*S. mutans*) is important in caries development because of its ability to synthesize extracellular polysaccharides (EPS) matrix.^4^ EPS, with all the oral microorganisms embedding in,^5^ is essential in oral microenvironment, because it can form a well-structured biofilm on the tooth surface.^1,6,7^ It can also function as a sensor for environmental signals and provide a long-term dynamic protection for microbial communities.^6,8^ Nowadays, with the thriving development of targeted therapies, regulations on EPS metabolism and biofilm formation have gained considerable attention.^9,10^ Nanoparticles have been used to disrupt biofilm and a small molecule which can selectively target *S. mutans* biofilms has also been reported to effectively reduce dental caries in vivo without affecting the overall oral microbiota.^11,12^ Researchers have found that the production, distribution as well as microcosmic characteristics of EPS all take a considerable part in the pathogenicity of polymicrobial biofilm.^13,14^ So, an understanding of specific characteristics of EPS derived from *S. mutans* biofilm will provide more solid evidences for caries prevention, which have not been elucidated yet.

VicRKX signal transduction system consists of three encoding proteins: VicK, a histidine protein kinase; VicR, a global response regulator (RR); and VicX, a putative hydrolase.^15–17^ VicK, as an essential link between the environmental stimuli and cellular status, is supposed to sense and transmit chemical signals to downstream regulatory proteins, like VicR and GcrR.^18,19^ And subsequently, it can regulate transcriptional levels of biofilm associated genes, like *gtfB/C*, *ftf* and *gbpB*.^20–22^ As a result, *vicK* gene possesses an important position in multiple bioactivities of *S. mutans*, particularly biofilm formation.^23,24^ What’s more, an epidemiological research has reported that the frequency of a *vicK* C470T missense mutation was higher in the high-severity caries group than in the caries-free group.^25^ The mutation of C470T contributes to the alternations of PAS domain of VicK protein, and PAS domain is a major sensor to environmental stresses,^26^ which means *vicK* gene, as well as VicK protein are associated with the cariogenicity of *S. mutans* closely.

At present, few studies on the relationship between microcosmic structures of EPS and cariogenicity of *S. mutans* have been found. Although *vicK* gene can modulate biofilm formation and virulence of *S. mutans* in some degree, it is not clear whether this modulation is related to EPS metabolism, especially its structural characteristics. And the comprehensive regulation among *vicK* gene, transcriptional levels of downstream genes, EPS synthesis and cariogenicity of *S. mutans* remains unknown. Hence, this study aimed to identify the role of *vicK* gene in the cariogenicity of *S. mutans* in vitro and in vivo, as well as to elucidate effects of *vicK* gene on the EPS.

## Results

### *vicK* was involved in biofilm morphological changes

**Fig. 1:**
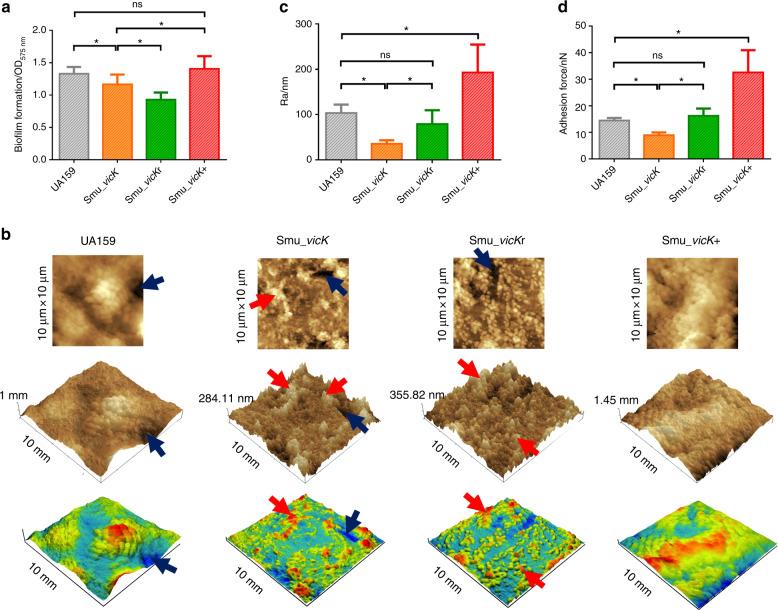
Analysis of biofilm morphologies. Unpaired *t* test was used to detect the statistical significances. **a** Biomass was quantified by CV staining, and the optical density at 575 nm was read. (*n* = 6; *P < 0.05; ns no significant difference). **b** Surface characteristics and the 3-D reconstruction of 24 h biofilms (an area of 10 μm × 10 μm, with a bar indicating vertical deviations) with the light (red) and dark (blue) zone representing peaks and valleys. **c** Surface roughness (Ra) of biofilms was obtained via AFM. (*n* = 4; *P < 0.05; ns no significant difference). **d** Adhesion force data were obtained from AFM. (*n* = 3; *P < 0.05; ns no significant difference)

To compare the abilities of different strains to form biofilms, crystal violet (CV) assay was used and the results showed that the mean value of *Smu_vicK* (the *vicK* deficient strain) biofilms was statistically lower than that of UA159 (Fig. 1a). Atomic force microscopy (AFM) observations indicated a peak-and-valley topography in Smu_*vicK* and *Smu_vicK*r (the *vicK* complementary strain) biofilms (peaks: red arrows; valleys: dark blue arrows; Fig. 1b). UA159, as well as the *Smu_vicK*+ (the *vicK* overexpression strain) biofilms had smoother and more flourishing surface (Fig. 1b). Mean Ra of *Smu_vicK* biofilms was markedly lower than the UA159 group, while that of *Smu_vicK*+ was significantly higher and that of *Smu_vicK*r was not statistically different (Fig. 1c). Adhesion force of *Smu_vicK* biofilms was statistically lower, whereas *Smu_vicK*+ appeared an enhanced capacity (Fig. 1d).

### *vicK* changed production and distribution of EPS in biofilm

**Fig. 2:**
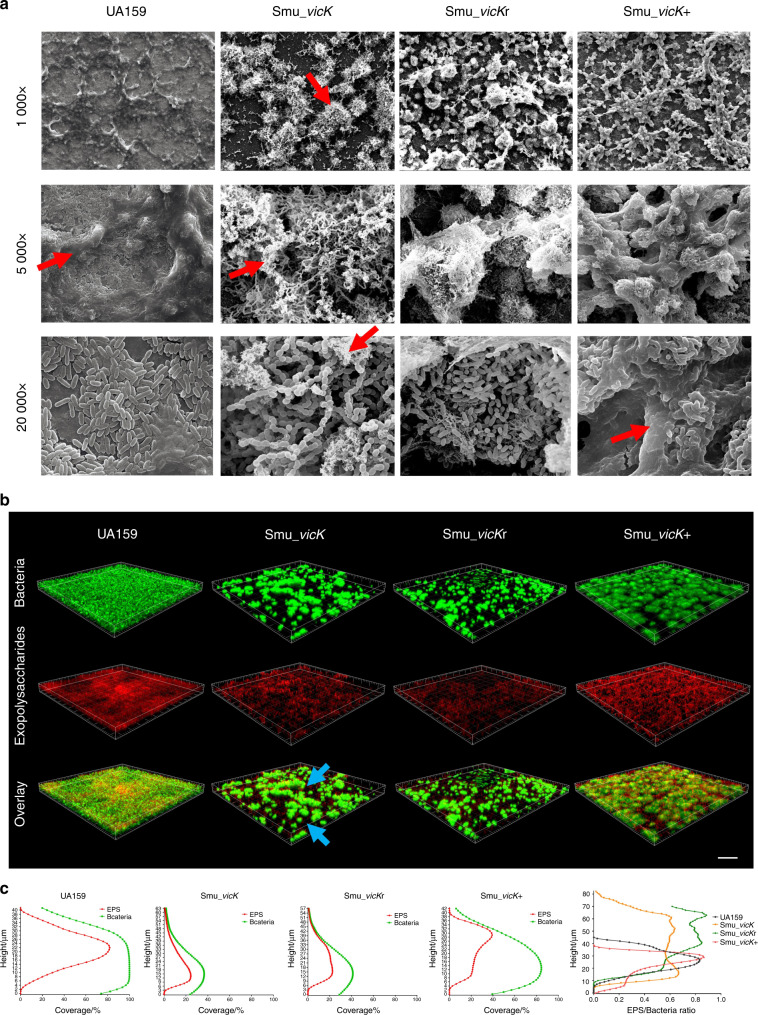
Analysis of 3-D bacteria-polysaccharides structure in mature biofilms of *S*. *mutans* strains. **a** Biofilm architecture was observed by SEM, in which images were taken at 1 000×, 5 000×, and 20 000× magnifications, respectively (EPS: red arrows). **b** Representations of glucans distribution and biofilm reconstructions were revealed by CLSM, where EPS stained with Alexa Fluor 647 were red and total bacteria stained with SYTO9 were green. Images were taken at 20× magnification and the scale bar indicated 100 μm. *Smu_vicK* biofilms showed small clusters of bacterial cells without enough EPS filling the space between them (light blue arrows). **c** Quantification of EPS and bacteria components, as well as EPS/bacteria ratios at different heights were performed with COMSTAT

Compared with UA159, *Smu_vicK* biofilms demonstrated an aberrant architecture with more scattered microcolonies and thinner EPS (Fig. 2a). Evidently, *Smu_vicK* biofilms seemed to be devoid of EPS when scanned under a higher magnification (5 000×). On the other hand, *Smu_vicK*+ cells were densely encased by enriched EPS (Fig. 2a). Confocal laser scanning microscopy (CLSM) data showed that only small clusters of bacterial cells were observed in *Smu_vicK* and *Smu_vicK*r biofilms and EPS were synthesized mostly around the cells, while there were much more EPS enmeshing the bacterial aggregates and filling the space between them in UA159 and *Smu_vicK*+ biofilms (Fig. 2b). As shown in Fig. 2c, both EPS and bacterial cells of *Smu_vicK* and *Smu_vicK*r biofilms were less than those of UA159 and *Smu_vicK*+ biofilms. EPS/bacteria ratios of the biofilm formed by *Smu_vicK* were significantly lower than those of UA159 and *Smu_vicK*r.

### Analyses of microcosmic structures of EPS generated from biofilms

**Fig. 3:**
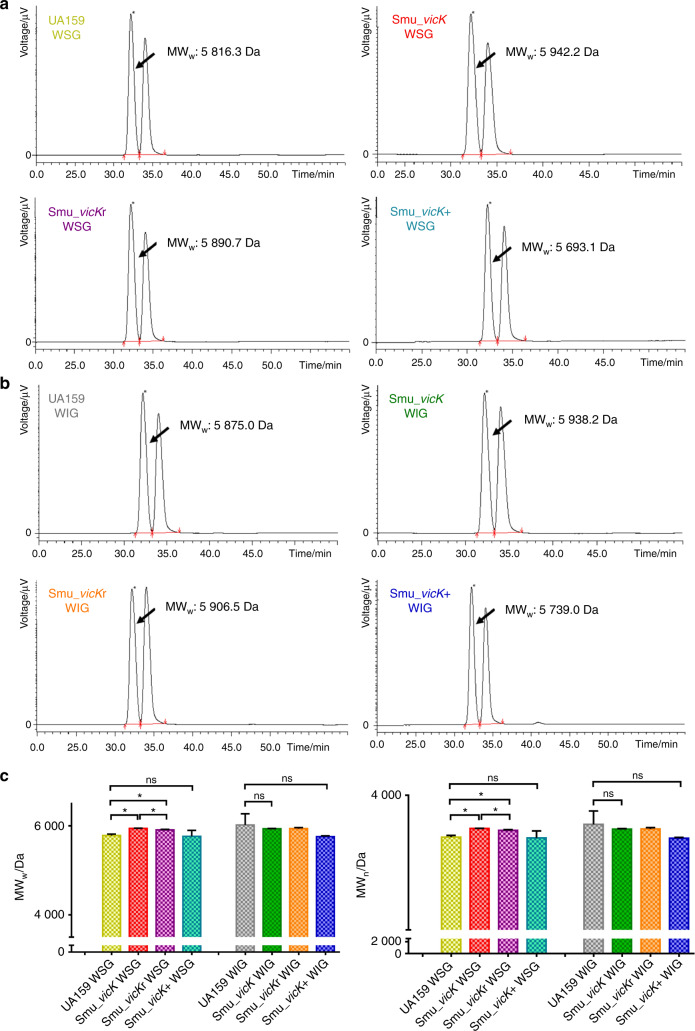
Molecular weight distributions of EPS were estimated by GPC. The calibration curves were constructed based on the molecular weight of standard dextran and retention time (RT), which were lgMW_w_ = −0.285 1RT + 12.969 and lgMW_n_ = −0.266 6RT + 12.261, with *R*^2^ = 0.994 5 and 0.993 9, respectively. **a**, **b** Typical GPC chromatograms of polysaccharides from WSGs and WIGs. **a** Representative MW_w_ for WSGs. **b** Representative MW_w_ for WIGs. **c** Distributions of mean MW_w_ and MW_n_. One-way ANOVA was used to detect the statistical significances. (*n* = 3; *P < 0.05; ns no significant difference)

MW_w_ (weight-average molecular weight) and MW_n_ (number-average molecular weight) were demonstrated in Fig. 3. Water-soluble glucans (WSGs) and water-insoluble glucans (WIGs) isolated from *Smu_vicK* biofilms presented the highest MW_w_ (5 942.2 Da and 5 938.2 Da, respectively), while those from *Smu_vicK*+ illustrated the lowest MW_w_ when UA159 was as a reference (Fig. 3a, b). For further analysis, the mean molecular weight of WSGs from *Smu_vicK* biofilms were statistically higher than those from UA159 biofilms (Fig. 3c).

**Fig. 4:**
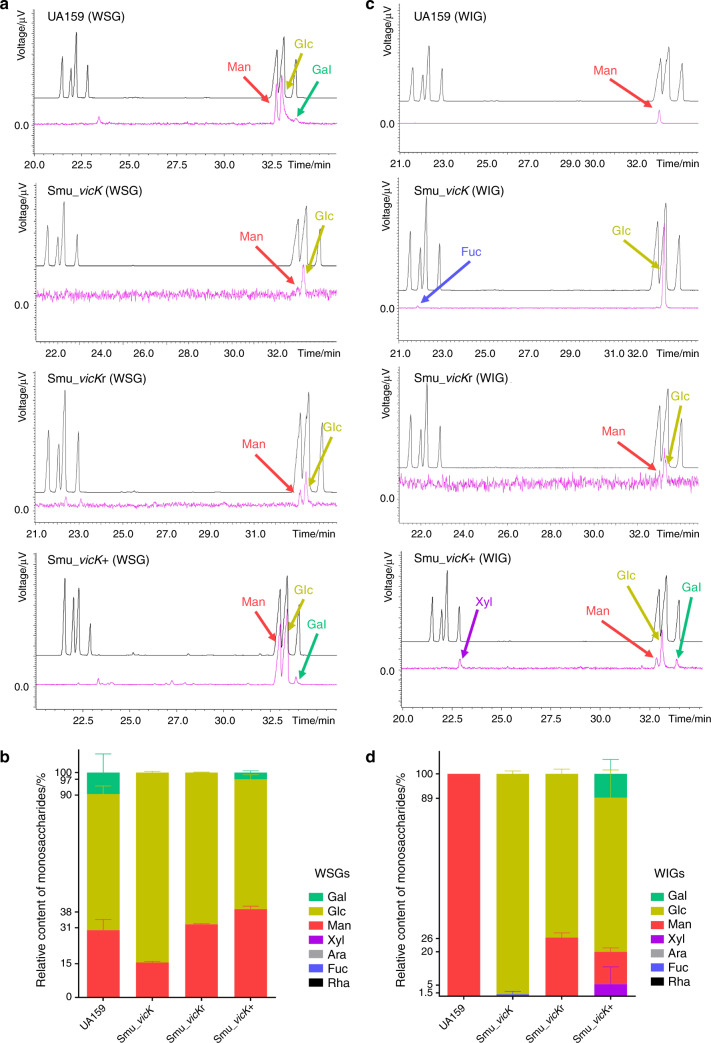
Monosaccharides of EPS were analyzed by GC‐MS. **a** Typical chromatograms of constituent monosaccharides in WSGs. In these profiles, the black image represented standard samples in sequence: Rha, Fuc, Ara, Xyl, Man, Glc, and Gal, while the pink one represented the experimental samples. **b** Molar ratios of different monosaccharides in WSGs. (*n* = 2). **c** Typical chromatograms of constituent monosaccharides in WIGs. **d** Molar ratios of different monosaccharides in WIGs. (*n* = 2)

Mannose and glucose were main constituents in both WSGs and WIGs (Fig. 4). Particularly, *Smu_vicK* WSGs consisted of lower mannose (15.55% ± 0.36%), higher glucose (84.46% ± 0.36%) and none galactose when compared to UA159 WSGs, in which mannose was 29.96% ± 3.33%, glucose was 60.49 ± 2.53%, and galactose was 9.56% ± 5.86% (*n* = 2, Fig. 4b). At the same time, WSGs from *Smu_vicK*r biofilms were composed of mannose (32.62% ± 0.14%) and glucose (67.38% ± 0.14%); WSGs from *Smu_vicK*+ biofilms were more like those from UA159 (Fig. 4a, b). For WIGs (Fig. 4c, d), UA159 exhibited a 100% content of mannose and *Smu_vicK* showed a 99.09% ± 0.91% content of glucose. Molar ratios of *Smu_vicK*r WIGs were 26.41% ± 1.49% and 73.59% ± 1.49%, for mannose and glucose, respectively. And *Smu_vicK*+ WIGs seemed to be more complicated consisting of xylose, mannose, glucose and galactose.

### Depletion of *vicK* represses cariogenicity of *S. mutans*

**Fig. 5:**
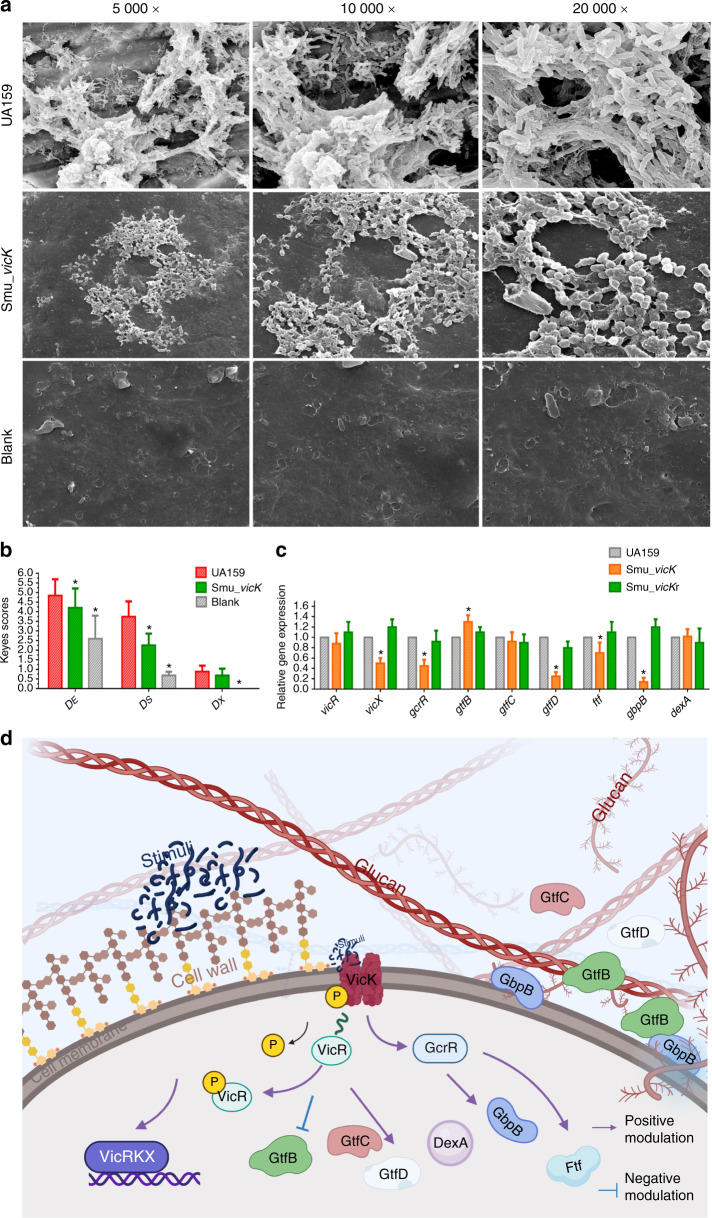
Influences of the deletion of *vicK* gene on cariogenicity and associated genes. **a** Dental plaques on mandibular molars in rats were shown by SEM with pictures taken at 1 000×, 5 000×, and 20 000× magnifications. **b** The degrees of caries were divided into three categories by depth of penetration: *D*_E_, *D*_S_, and *D*_X_ according to the modified Keyes scores. Unpaired *t* test was used to detect the statistical significances. (*n* = 10; *P < 0.05). **c** Expressional levels of EPS-correlated genes were measured by qRT-PCR, where UA159 was taken as the control group and the *gyrA* gene was used as an internal standard (not shown). One-way ANOVA was used to detect the statistical significances. (*n* = 3; *P < 0.05). **d** The schematic illustration of regulations conducted by *vicK* on the EPS metabolism of *S. mutans*. Created with BioRender.com. Adapted from “ECM (Extracellular Matrix)”, by BioRender.com (2021). Retrieved from https://app.biorender.com/biorender-templates. After being activated by environmental stimuli, VicK has vital influences on the expression of genes correlated with EPS, whose encoding proteins promote the synthesis of EPS, subsequently enhances the biofilm virulence

Cariogenicity of *Smu_vicK* in vivo was examined by a specific pathogen-free (SPF) rat model. Biofilm overview and EPS distribution were captured by scanning electron microscopy (SEM). In SEM images, *Smu_vicK* biofilms were sparser with thinner EPS than those formed by UA159 (Fig. 5a). According to the severity and penetration of caries, dental caries was classified into three categories: enamel only (*D*_E_), slight dentinal (*D*_S_) and extensive dentinal (*D*_X_). *Smu_vicK* group exhibited a significantly increased degree of caries compared to blank group and a significantly decreased degree of caries compared to UA159 group in the *D*_E_ and *D*_S_ categories (Fig. 5b). However, the severity of *D*_X_ was unaffected by depletion of *vicK* gene.

### *vicK* alters the expressional levels of virulence-related genes

To evaluate effects of *vicK* gene on signal transduction system and cariogenicity-related genes, the *gyrA* gene was used as an endogenous reference. As shown in Fig. 5c, transcriptional levels of *vicX* and *gcrR* genes were decreased in *Smu_vicK* when compared to UA159. Simultaneously, *gtfD*, *ftf*, and *gbpB* were also downregulated in *Smu_vicK*. On the contrary, the expressional level of the *gtfB* gene was significantly increased in *Smu_vicK*. No difference was observed in the expressions of *gtfC* and *dexA*.

## Discussion

Biofilms and EPS are inseparably linked. Biofilms develop when microorganisms accumulate on tooth surfaces and secret EPS.^27^ At the same time, EPS initiates the adhesion for first colonizers and is critical for dental biofilms development.^28^ Working together, they can be virulent and cause infectious diseases, like dental caries.^29^ In our study, *Smu_vicK* showed a reduced ability in biofilm accumulation, which was consistent with previous studies that found *Smu_vicK* biofilm clumpy and deficient in microcolonies formation.^23,30^ Biofilms can act as a barrier and cause resistance against harmful factors, and thicker they are, more protective they are for microbes within.^31^ Thin biofilms like *Smu_vicK* biofilms might be less stable and more easily disrupted, which was consistent with what Senadheera had reported before.^23^ AFM observation of *Smu_vicK* biofilms demonstrated a bumpy topography with spike-like peaks, indicating that knockout of *vicK* gene yielded a thin biofilm with less EPS. Biofilm development is a complex process including cell to cell adhesion. Physical or molecular interactions are involved in this process.^32^ It also has been reported that the surface roughness does not necessarily affect bacterial adhesion.^23^ The limitation of this study was that we did not measure the surface roughness of a substratum surface but the surface roughness of the biofilm itself. Interestingly, *Smu_vicK* biofilms were thicker than those of UA159 as shown in Fig. 2c, but they seemed sparser and with less EPS (Fig. 2b). In present study, the biofilm surface roughness and adhesion force of *Smu_vicK* biofilms were declined, indicating biofilm surface roughness and adhesion force may be reflections of EPS production in *S. mutans*, which also warranted further investigations.^23,33^ Similar results came out in SEM and CLSM observations. It was worth mentioning that EPS of *Smu_vicK* biofilm did overlap with the microcolonies but it was few. Previous reports also showed that the *vicK*-deficient strain had a drastically reduced glycolytic rates.^33^

Indeed, construction of the *vicK* complementary strain was limited in this study, where *vicK* expression was slightly higher than *Smu_vicK* but not close to UA159 level (Fig. S1). As a result, *Smu_vicK*r biofilms were thin and more like *Smu_vicK* biofilms. One possible explanation was that the introduction of an exogenous plasmid vector might interfere with the intracellular homeostasis and growth of the mutants, which has been reported in our previous study.^16^ Although *Smu_vicK*r biofilms show less biomass than the one of *Smu_vicK* biofilms, the phenotypes of roughness and adhesive forces in the *Smu_vicK*r biofilms were partially restored when compared to those of UA159 biofilms. *Smu_vicK*+ biofilms did exhibit a slight increase in biomass or EPS when compared to those of UA159. We speculated that the difference was not that evident because of the introduction of the plasmid.^16^ Biofilms of *Smu_vicK*+ were not as mature as UA159 in Fig. 2, but it also showed EPS/bacteria ratios of *Smu_vicK*+ biofilms were higher than those of UA159. One possible explanation for *Smu_vicK*+ biofilms not as mature as UA159 was that *Smu_vicK*+ biofilms had larger microorganism clusters and more EPS.

Taken together, deletion of *vicK* gene not only impaired biofilm formation, but also repressed EPS production. EPS can encapsulate microbial communities and provide enough attachment sites for them to form structured biofilms, contributing to biofilm lifestyle and virulence.^34^ In addition to EPS, bacterial aggregation is also an essential part in biofilm formation.^23^ We found a rough bacterial aggregation of *Smu_vicK* in SEM, an evidently longer chain length and a larger colony of *Smu_vicK*+ (Fig. 2 and Fig. S2). Decreased EPS and aberrant biofilm might result in alteration of cariogenicity of *S. mutans*.

Exopolysaccharides is part of macromolecules within extracellular matrix. Both WSGs and WIGs are of great significance. WSGs is able to provide energy source; WIGs can provide protective shelters for the biofilms and modulate environmental acidification.^35–37^ Targeting EPS is a promising approach in biofilm control and recent studies have declared kinds of nanoparticles disrupting biofilm microenvironment, which allows disruption of the matrix.^38–40^ Targeted therapy for dental caries is becoming more popular,^41^ so a thorough understanding of the targeted EPS is a keystone. Structural features of polysaccharides like molecular weight may play an important role in various biological activities.^42,43^ For example, a high-molecular-weight polysaccharide fraction from *Sparassis latifolia* is recognized to account for a limited biological activity because it makes the fungus difficult to adhere or penetrate cell membranes.^44,45^ Combined with the data we got, it was speculated that the upregulation of molar mass in *Smu_vicK* EPS might be relevant to its impairment. Molecular weight of polysaccharides can be influenced by glycoside linkage, which is attributed to the linkage cleavage during the enzymatic degradation.^46^ Fructosyltransferase (Ftf, coded by *ftf*) converts sucrose into a predominantly β2,1-linked fructan polymer.^47^ Glucosyltransferases (Gtfs, encoded by *gtfB/C/D* genes) are also linkage-related, in which GtfB synthesizes α1,3-glycosidic linkages of WIGs and GtfD synthesizes α1,6-glycosidic linkages of WSGs.^48^ We assumed the altered expressional levels of *ftf* and *gtfB/D* genes in *Smu_vicK* (Fig. 5c) probably induced glycoside linkage alternation and subsequent molecular weight of polysaccharides. Further studies like methylation analysis will provide the information of the glycosidic linkages of EPS.^49^ Monosaccharides are essential substrates to exopolysaccharide chains, and different compositions of them can make different biofilm matrix.^50,51^ Constituent ratio of glucose experienced evident increasing in EPS of *Smu_vicK* biofilms, indicating a rising capability of *Smu_vicK* to synthesize glucose. It was speculated that polysaccharides of *Smu_vicK* biofilms were heteropolysaccharides containing glucose, and the side chains might be composed of mannose or fucose, which can be confirmed by connection mode analysis in further studies.^52^ EPS is a vital attribute for the bacterial community structuring and contributes a lot in biofilm lifecycle and subsequent caries development.^53^ We regarded that *vicK* gene and microcosmic structures of EPS were closely related, which might have a relationship with cariogenicity of *S. mutans* biofilms.

A dysregulation of biofilm formation and decreased cariogenicity of *Smu_vicK* in vivo could be seen in this study. Evidently, these results were consistent with the results in vitro. However, Senadheera had found that in low-cariogenic diet feed rats, the development of dentinal lesions was not significantly altered in *Smu_vicK* group compared with UA159,^23^ which was different from what we found when we fed the rats a normal diet with drinking water containing 2% sucrose. *S. mutans* can make effective use of dietary sucrose for EPS production, using GtfB/C/D.^54,55^ Both the diet and the nature of EPS can determine the diffusion properties of plaque and cariogenicity of *S. mutans*. VicK acts as a receptor of extracellular stimuli,^56^ and when it was deleted, the capacity of bacterial cell to get transduction signals might be impaired. On the other hand, expressions of *gtfB/C/D* genes could also be affected by *vicK* as previously reported,^23^ which are associated with sucrose. One possible explanation of this difference was that *Smu_vicK* had defects in biofilm formation while UA159 formed more compact and cariogenic biofilms under high-cariogenic circumstances. In addition, it has been found that the *vicK*-deficient strain produced less lactic acid,^33^ which might partially contribute to the low cariogenicity of the *Smu_vicK* biofilm. These data suggested that we should attach more importance to *vicK* gene and dietary sucrose.

In present study, *vicK* gene positively modulated *vicR/X* and *gcrR*. The *vicR/K/X* gene are in the same operon: the *vic* operon,^57^ so deletion of *vicK* gene might influence the transcriptional levels of *vicR/X*. GcrR (also named CovR, coded by *gcrR* gene) is known as an orphan response regulator in *S. mutans*, with no exclusively linked cognate histidine kinase.^58^ There is a co-regulation between GcrR and VicRK,^59,60^ in which, like VicR, GcrR can also directly binds to the promoter regions of some genes, but opposite to VicR, it acts like a repressor.^61–63^ And VicK can activate GcrR by phosphorylating it.^64,65^ The declined expressional level of *gcrR* in this study was not in coincidence with the opposite mechanism between VicR and GcrR. What’s more, we found a significant decrease of GcrR protein in *Smu_vicK* (Fig. S3). Although there was also a decrease in expressional level of VicR protein, it seemed relatively stable when compared with GcrR (Fig. S3). On one hand, it has been reported that deletion of *vicK* gene does not have a significant influence on the ratio of phosphorylated VicR to unphosphorylated VicR.^66^ On the other hand, it has also been authenticated that neither dimerization nor phosphorylation status of GcrR are essential for its activity.^59^ As a result, we speculated that VicK might also influence the expressional level of GcrR in some way to balance the regulatory system, which need further studies. Simultaneously, *gtfD*, *ftf* and *gbpB* were also downregulated in *Smu_vicK* as previously reported.^23^ The less-cariogenic biofilm with poorer EPS generated by *Smu_vicK* might be resulted from the defect of *ftf* and *gbpB* genes because Ftf and GbpB (encoded by *gbpB*) should have enhanced the cariogenic potential of *S. mutans* by extending acidification or working with Gtfs.^67–69^ Unexpectedly, the expressional level of *gtfB* gene was significantly increased and that of *gtfC* was unchanged in *Smu_vicK*, which were in contrast with a previous observation showing a 3.0 and 1.4-fold downregulation of *gtfB*/*C* in *Smu_vicK* at mid-log phase grown in brain heart infusion (BHI) medium.^70^ We considered the variation in nutrients was responsible for the differences.^71,72^ It was interesting that the increase of *gtfB* did not appear to provide a mechanically integrated and stable extracellular insoluble matrix for *Smu_vicK* biofilms. This phenomenon might be explained by the extreme decrease of *gtfD*, whose products are supposed to serve as primers for GtfB activity.^73^ In *S. mutans*, Gtfs perform a concerted action to produce glucans and affect EPS.^74,75^ It was once discussed that an optimal GtfB/GtfC/GtfD ratio was necessary for appropriate colonization of *S. mutans* in vitro.^76^ We speculated that the expression of *vicK* gene mostly influenced EPS-synthesis genes in a positive way because the expressional level of *dexA* gene was not changed in *Smu_vicK*. A schematic illustration was shown in Fig. 5d. Understanding the mechanisms involved in biofilm-associated gene expressions is crucial for us to develop new therapeutic strategies. It has been reported that expressional levels of *vicR/gcrR* genes, key regulators to EPS-synthesis, are maximal in *S. mutans* which are at mid-exponential phase.^77^ However, the gene expressions of planktonic cultures may not reflect the expressions of biofilm grown cells, which needs further investigations.

Carbohydrate metabolism is necessary to viability and virulence of *S. mutans*, in which production of lactic acid, acid tolerance and production of EPS are all essential.^78^ *S. mutans* is able to produce lactic acid as a metabolic product, which induces lower pH value in environment, as well as the enamel erosion and dental caries. The acid tolerance response becomes a key survival mechanism for the acid stress adaption.^79^ Senadheera et al. had studied the effects of *S. mutans vicK* gene on acidogenicity and aciduricity in the presence of glucose. They found *vicK* deletion mutant produced less lactic acid with its acid tolerance enhanced.^33^ However, the effects of *S. mutans vicK* gene on EPS production in the presence of sucrose were not fully elucidated. So, in current study, we provided evidences proving the modulation of *vicK* gene on EPS of *S. mutans* biofilm. The *vicK* gene regulated biofilm characteristics, including synthesis as well as component and structural modifications of EPS. These data illustrated a significant situation where *vicK* was a promising governor, being involved in cariogenicity of *S. mutans* through EPS metabolism. We hypothesized *vicK* gene enhanced transcriptional levels of EPS-induced genes in a signal transduction cascade and it could increase the production of EPS, change the microcosmic features of EPS as well as strengthen the virulence of *S. mutans*.

## Materials and methods

### Strains and bacterial culture conditions

*S. mutans* standard strain,^36^ UA159 (Table 1) was provided by State Key Laboratory of Oral Diseases, West China School of Stomatology, Sichuan University. An erythromycin resistance cassette was utilized to knock out the *vicK* gene in UA159 chromosome by polymerase chain reaction (PCR) ligation mutagenesis to get *Smu_vicK*.^23,80^ Recombinant pDL278 plasmids which contained the *vicK* gene coding region were introduced into *Smu_vicK* and UA159 cells respectively to construct the *vicK* complementary strain (*Smu_vicK*r) and the *vicK* overexpression strain (*Smu_vicK*+).^81^ The expressions of *vicK* gene in the mutants were verified by quantitative real-time PCR (qRT-PCR) (Fig. S1). Conventionally, UA159, *Smu_vicK*, *Smu_vicK*r, and *Smu_vicK*+ were cultured into mid-exponential phase in BHI medium (Becton, Dickinson and Company, Sparks, MD 21152 USA) at 37 °C, anaerobically, with appropriate antibiotics when necessary. The achieved final concentrations of antibiotics were 10 μg·mL^−1^ erythromycin for *Smu_vicK*, 1 000 μg·mL^−1^ spectinomycin for *Smu_vicK*+, 10 μg·mL^−1^ erythromycin and 1 000 μg·mL^−1^ spectinomycin for *Smu_vicK*r.

**Table 1:** Bacterial strains and plasmid used in this study

Strain or plasmid	Description	Source of reference
UA159	*S. mutans* UA159	ATCC 700610
*Smu*_*vicK*	*S. mutans* UA159 with in-frame replacement with an erythromycin cassette	This study
*Smu*_*vicK*r	*Smu_vicK* transformed with pDL278 encoding *vicK* gene	This study
*Smu*_*vicK* +	*S. mutans* UA159 transformed with pDL278 encoding *vicK* gene	This study
pDL278	*Escherichia coli*-*Streptococcus* shuttle vector and expression plasmid (spectinomycin)	Gifted by Dr. Huichun Dong, Institute of Microbiology, Chinese Academy of Sciences

### Biofilm formation

Strains were grown to mid-exponential phase at an OD_600 nm_ of 0.5 and then were diluted 1:100 into BHI medium containing 1% (w/v) sucrose (BHIS). Biofilms were formed at 37 °C, anaerobically for 24 h.

### CV assay

Biofilm mass was quantified by CV assay as previously described.^82^ Briefly, biofilms formed on a polystyrene surface in a 96-well plate were washed by sterilized deionized distilled water three times to remove planktonic bacteria cells, then were stained with 0.1% (w/v) CV for 15 min. The stained biofilms were gently rinsed again and air dried, followed by an addition of 33% (v/v) glacial acetic acid to solubilize the dye. At last, the optical density was read at 575 nm (SpectraMax^®^ iD5, Molecular Devices, San Jose, California, USA).

### Atomic force microscopy

Surface topography and adhesion force of the biofilms were analyzed by AFM as conducted previously.^83,84^ Biofilms were incubated in a 24-well plate for 24 h with round, sterile glass slides, then the slides were washed by sterilized deionized distilled water and dried for two minutes in air. Shimadzu SPM-9700 system (SHIMADZU, Kyoto, Japan) was conducted in the contact mode using a specialized probe (HYDRA-ALL-G-20, APPNANO, USA) for the measurements.

### Scanning electron microscopy

Architecture of the biofilms was measured by SEM (Inspect F, FEI, Eindhoven, Holland) using round, sterile glass slides.^85^ The biofilms on slides were washed by sterilized deionized distilled water then fixed with 2.5% (v/v) glutaraldehyde overnight. Next day, they experienced a sequential dehydration in ethanol solutions and then were prepared for imaging.^86^ The specimens were examined at 1 000×, 5 000×, and 20 000× magnifications, and representative pictures are shown.

### Confocal laser scanning microscopy

Three-dimensional (3-D) structure and EPS distribution of the biofilms were observed by CLSM (Olympus FV1000, Japan).^87^ 1 μmol·L^−1^ Alexa Fluor 647 (Invitrogen, Eugene, Oregon, USA) were added into 2 mL BHIS with 20 µL bacterial cultures before biofilm incubation to label dextran conjugate. Biofilms were incubated in a 24-well plate for 24 h in dark, with round, sterile glass slides. After biofilm formation, nucleic acid of *S. mutans* cells were labeled with 2.5 μmol·L^−1^ SYTO9 (Invitrogen, Eugene, Oregon, USA). Microscopic observations were performed with a 20× objective lens, and 3-D images were reconstructed by Imaris 7.0.0 software (Bitplane, Zürich, Switzerland). Calculation of EPS/bacteria biomass were performed with COMSTAT.

### Exopolysaccharides assessment

Molecular weights of EPS were analyzed by gel permeation chromatography (GPC) and monosaccharide composites were analyzed by gas chromatography–mass spectrometry (GC–MS). Both of WSGs and WIGs derived from 24 h biofilms were isolated and purified for GPC and GC–MS analysis as reported before.^16^ In short, biofilms suspended by sterilized deionized distilled water were centrifuged to separate WSGs solution and other insoluble precipitates containing WIGs. The insoluble precipitates were dissolved in 1 mol·L^−1^ NaOH solution and then filtered through a 0.22 μm filter (Merck Millipore Ltd. Tullagreen, Carrigtwohill, Co. Cork, IRL) to get WIGs solution. Then, 20% (w/v) trichloroacetic acid was added into the solution mentioned above for protein precipitations. After that, solutions were dialyzed (cellulose membrane with a molecular weight cut-off of 500–1 000 Da) for further purification and were lyophilized afterwards. The lyophilized powder was used for GPC and GC-MS.^88^ For GPC analysis, sample polysaccharides in a final concentration of 5 mg mL^−1^ and standard solutions of dextran in different molecular weights (MW; MW 1 152, 5 200, 11 600, 148 000, 273 000, 410 000 Da; Waters, Massachusetts, USA) were loaded in a high performance liquid chromatograph (LC-10A, SHIMADZU, Japan).^51^ For GC–MS analysis (QP 2010 Plus, SHIMADZU, Japan), samples were further acetylated and the compositions of EPS were identified with standard monosaccharide substances (Sigma, San Francisco, CA, USA), which included rhamnose (Rha), fucose (Fuc), arabinose (Ara), xylose (Xyl), mannose (Man), glucose (Glc), galactose (Gal).^89^

### Cariogenicity ability in vivo

Effects of *vicK* gene on cariogenicity of *S. mutans* in vivo were assessed in a SPF rat model.^90^ This study was approved by the Ethics Committee of West China Hospital of Stomatology, Sichuan University (NO: WCHSIRB-D-2019-144), and conducted in IVC Experimental Animal Center of Public Health, Sichuan University (Chengdu, China). Thirty caries-susceptible, Osborne–Mendel rats (15 male rats and 15 female rats), aged 14 day old (Dashuo Company, Chengdu, China) were included and randomly assigned into 3 groups: Blank (the negative control), UA159 (the positive control), and *Smu_vicK* (the experimental group). Posterior to endogenous Streptococci test,^91^ on days 23–30, rats were infected orally, daily for one week, using 200 μL bacterial suspensions that comprised UA159 or *Smu_vicK*, respectively. Bacterial suspensions were collected when strains grew to mid-exponential phase at the same OD_600nm_ 0.4. All rats received drinking water containing 2% sucrose as well as a normal diet, and animals were sacrificed on day 50. Then, their lower jaws were excised for plaque observation using SEM and caries level determination using modified Keyes score.^92^

### Gene expression assay

*S. mutans* cells, which included UA159, *Smu_vicK* and *Smu_vicK*r, were collected at mid-exponential phase in BHIS by centrifugation at 4 000 r·min^−1^ for 15 min (Thermo Fisher SCIENTIFIC, Germany). Gene expression was analyzed by qRT-PCR. Total RNAs of *S. mutans* cells were isolated and purified using MasterPure^TM^ Complete DNA and RNA Purification kit (Lucigen Corporation, Wisconsin, USA) according to the instructions. Concentration and purity of total RNAs were assessed by NanoDrop One^C^ Microvolume UV–-Vis Spectrophotometer (Thermo Scientific, Waltham, MA, USA). After that, RNAs were reverse transcribed into cDNA using PrimeScript^TM^ RT reagent kit with gDNA eraser (TAKARA BIO INC. Kusatsu, Shiga, Japan). Optimal sample or primer concentrations were followed by the instruction of TB Green^TM^ *Premix Ex Taq*^TM^ II kit (TAKARA BIO INC. Kusatsu, Shiga, Japan). In brief, each 20 μL reaction mixture included 10 μL of TB Green^TM^ *Premix Ex Taq*^TM^ II, 0.8 μL of 10 μmol·L^−1^ PCR Forward Primer, 0.8 μL of 10 μmol·L^−1^ PCR Reverse Primer, 2.0 μL of template cDNA and 6.4 μL of deionized water. And qRT-PCR was conducted in a LightCycler^®^ 480 System (Roche, Basel, Switzerland) according to the instruction above.^93^ The expressional levels of *gyrA*, *vicR/X*, *gcrR*, *gtfB/C/D*, *ftf*, *gbpB* and *dexA* genes were evaluated. All primers for qRT-PCR were designed based on the sequence of the *S. mutans* UA159 genome according to previous studies and obtained commercially (Sangon Biotech, Shanghai, China).^23,88^ They were listed in Table 2. Gene expressions were calculated by the 2^−ΔΔCT^ method in which *P* < 0.05 were thought to be significantly different.^94^

**Table 2:** List of the primers used in this study

Primer	Nucleotide sequence	Annealing temperature/°C	Size/bp
*PCR-ligation mutagenesis*			
erm-PF	5′ GGCGCGCCCCGGGCCCAAAATTTGTTTGAT 3′	52.3	876
erm-PR	5′ GGCCGGCCAGTCGGCAGCGACTCATAGAAT 3′		
*vicK*-P1	5′ TGGTAAAGCAGTATCTGGCGAGG 3′	53.1	886
*vicK*-P2	5′ GGCGCGCCATAGTGAGGAAGGCGAAGGGTC 3′		
*vicK*-P3	5′ GGCCGGCCCCAGGGACTTGATTCAAACACATTAG 3′	54.1	666
*vicK*-P4	5′ GGCTAAGGAAGGTTATGACACG 3′		
*primers for qRT-PCR*			
*gyrA*-F	5′ ATTGTTGCTCGGGCTCTTCCAG 3′	62	105
*gyrA*-R	5′ ATGCGGCTTGTCAGGAGTAACC 3′		
*vicR*-F	5′ CGCAGTGGCTGAGGAAAATG 3′	53	157
*vicR*-R	5′ ACCTGTGTGTGTCGCTAAGTGATG 3′		
*vicK*-F	5’ CACTTTACGCATTCGTTTTGCC 3′	52	102
*vicK*-R	5′ CGTTCTTCTTTTTCCTGTTCGGTC 3′		
*vicX*-F	5′ TGCTCAACCACAGTTTTACCG 3′	51.4	127
*vicX*-R	5′ GGACTCAATCAGATAACCATCAGC 3′		
*gcrR*-F	5′ ACCAGAGATGGACGGGTATG 3′	55	120
*gcrR*-R	5′ CACGATAGGTAGTGTCATTTTTAG 3′		
*gtfB*-F	5′ ACACTTTCGGGTGGCTTG 3′	49	127
*gtfB*-R	5’ GCTTAGATGTCACTTCGGTTG 3′		
*gtfC*-F	5′ CCAAAATGGTATTATGGCTGTCG 3′	50.5	136
*gtfC*-R	5′ TGAGTCTCTATCAAAGTAACGCAG 3′		
*gtfD*-F	5′ AATGAAATTCGCAGCGGACTTGAG 3′	55	245
*gtfD*-R	5′ TTAGCCTGACGCATGTCTTCATTGTA 3′		
*ftf*-F	5′ ATTGGCGAACGGCGACTTACTC 3′	52.8	103
*ftf*-R	5’ CCTGCGACTTCATTACGATTGGTC 3′		
*gbpB*-F	5′ AGCAACAGAAGCACAACCATCAG 3′	55	150
*gbpB*-R	5′ CCACCATTACCCCAGTAGTTTCC 3′		
*dexA*-F	5′ AGGGCTGACTGCTTCTGGAGT 3′	55	142
*dexA*-R	5′ AGTGCCAAGACTGACGCTTTG 3′		

### Statistical analysis

Statistical analysis was executed by SPSS 16.0 (SPSS Inc, Chicago, IL, USA). Unpaired *t* test and one-way ANOVA were utilized to detect the statistical significance. Generally, the differences between the means of study data were statistically significant if *P* < 0.05.

## Supplementary information

Supporting Information
