# Supplementary material for: The vicK gene of Streptococcus mutans mediates its cariogenicity via exopolysaccharides metabolism
Source: Int J Oral Sci. 2021 Dec 16;13:45. doi: 10.1038/s41368-021-00149-x (PMC8677823; doi:10.1038/s41368-021-00149-x)

**Supporting Information**

**Figure S1. Identification of the *vicK* gene expression in UA159, *Smu_vicK*, *Smu_vicK*r and *Smu_vicK*+ by qRT-PCR.** Cells were collected at mid-exponential phase grown in BHI and *gyrA* gene was the reference (not shown). *Smu_vicK*: (1.47±0.01) ×10^-4^; *Smu_vicK*r: (3.90±0.25) ×10^-3^; *Smu_vicK*+: 2.36±0.30. **P*<0.05


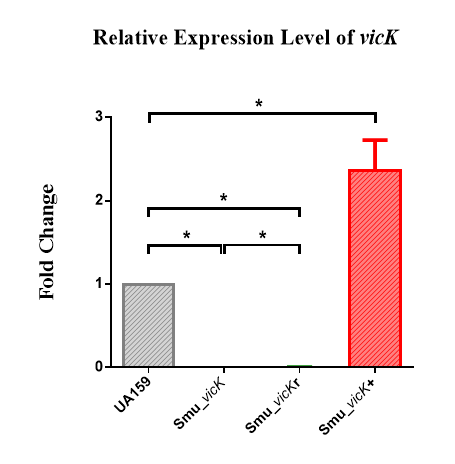


**Figure S2. Bacteria cell structures captured by SEM.** 3h biofilms of the parental and mutant stains built in BHIS were observed at 1000×, 5000×, and 50000× magnifications.


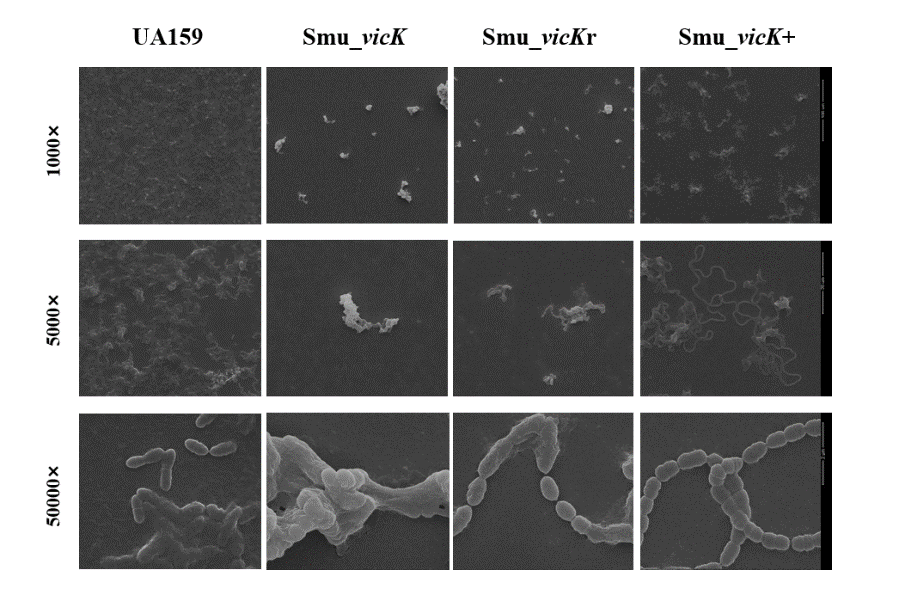


**Figure S3. Expressional level of VicR and GcrR proteins by Western Blot.** Cells were collected at mid-exponential phase grown in BHIS.


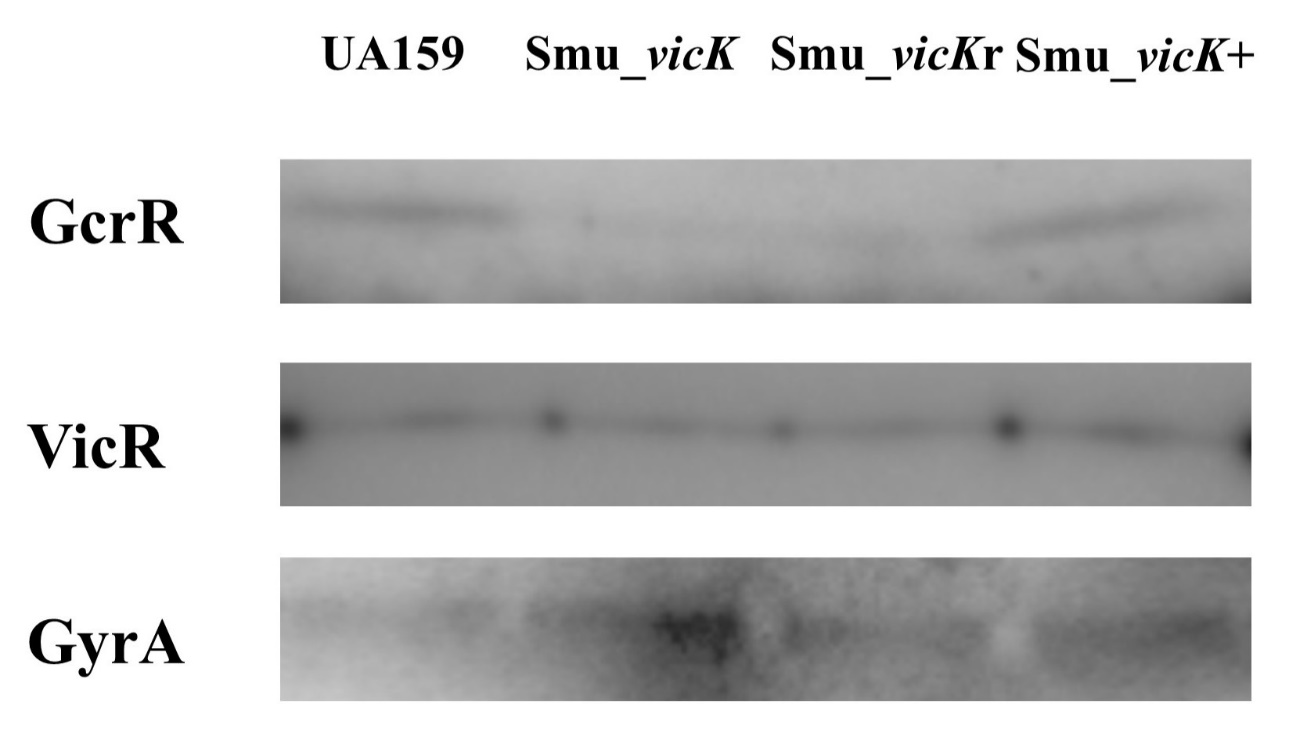

Supplement: Supplementary file 1 — Supporting Information [file 41368_2021_149_MOESM1_ESM.docx]
